# Development of a highly sensitive method for detection of JAK2V617F

**DOI:** 10.1186/1756-8722-4-40

**Published:** 2011-10-10

**Authors:** Anna H Zhao, Rufei Gao, Zhizhuang J Zhao

**Affiliations:** 1 Department of Pathology, University of Oklahoma Health Sciences Center, Oklahoma City, Oklahoma 73104, USA; 2 Oklahoma School of Science and Mathematics, Oklahoma City, Oklahoma 73104, USA; 3 Edmond H. Fischer Signal Transduction Laboratory, College of Life Sciences, Jilin University, Changchun, China

**Keywords:** *Tyrosine kinase*, myeloproliferative neoplasms, *JAK2*, *mutation*, *detection*, *diagnosis*

## Abstract

**Background:**

Ph- myeloproliferative neoplasms (MPNs) represent a heterogeneous group of chronic diseases characterized by increased expansion of hematopoietic cells of the myeloid lineage. JAK2V617F, an activation mutation form of tyrosine kinase JAK2, is found in the majority of patients with MPNs. Studies have demonstrated that JAK2V617F can cause MPNs, and various methods have been developed to detect JAK2V617F for diagnostic purposes. However, a highly sensitive method is still needed for the earliest possible detection and for disease prevention and treatment.

**Methods:**

In the present study, we developed a method dubbed restriction fragment nested allele-specific PCR (RFN-AS-PCR). The method consists of three steps: 1) initial amplification of DNA samples with PCR primers surrounding the JAK2V617F mutation site, 2) digestion of the PCR products with restriction enzyme BsaXI which only cleaves the wild type allele, and 3) detection of JAK2V617F by allele-specific PCR with nested primers.

**Results:**

We tested the sensitivity of the method by using purified plasmid DNAs and blood cell DNAs containing known proportions of JAK2V617F. We were able to detect JAK2V617F with a sensitivity of 0.001%. We further analyzed blood cell DNA samples from 105 healthy donors with normal blood cell counts and found three JAK2V617F-positive cases, which would have remained undetected using a less sensitive method.

**Conclusions:**

We have developed a highly sensitive method that will allow for detection of JAK2V617F at a very early stage. This method may have major implications in diagnosis and prevention of MPNs and related diseases.

## Background

Ph- myeloproliferative neoplasms (MPNs) represent a group of chronic conditions including polycythemia vera (PV), essential thrombocythemia (ET), and primary myelofibrosis (PMF) [1,2]. MPNs mainly affect older people with an average age of onset of 55 years. So far, there is no effective cure for the diseases. Complications associated with MPNs include thrombosis, hemorrhage, myeloid metaplasia, and acute leukemia. In addition, these diseases cause strokes and heart attacks that are usually fatal. The major molecular lesion in these diseases is JAK2V617F, which occurs in approximately 96% of PV, 65% of PMF, and 55% of ET cases [3-7]. Studies have demonstrated that transgenic expression or knock-in of JAK2V617F in mice causes MPN-like phenotypes [8-14]. JAK2V617F has thus become a valuable marker for diagnosis of MPNs and an excellent target for therapeutic drug development [2]. Several qualitative and quantitative techniques have already been developed for the detection of JAK2V617F. Results of JAK2V617F mutation assessment often depend both on the sensitivity of the employed method and the type of sample to be analyzed. Current methods for JAK2 genotyping include conventional sequencing, pyrosequencing, allele-specific PCR (AS-PCR), restriction fragment length polymorphism, real-time PCR, DNA-melting curve analysis, denaturing high performance liquid chromatography, and mass spectrometry [15-26]. These methods have reported sensitivities ranging from 0.01% to 5%, and each has its own advantages and disadvantages [27-29]. Some are not sensitive enough and yield ambiguous results, while others are sensitive but give nonspecific false positives. Also, some of these methods are labor-intensive and time-consuming, and they may require specialized or costly equipment and reagents. A more reliable and more sensitive method is still needed for the earliest possible detection of JAK2V617F, which will have major implications in diagnosis and prevention of MPNs.

## Results and Discussion

### Development of restriction fragment nested allele-specific PCR (RFN-AS-PCR), an improved method for detection of JAK2V617F

**Figure 1:**
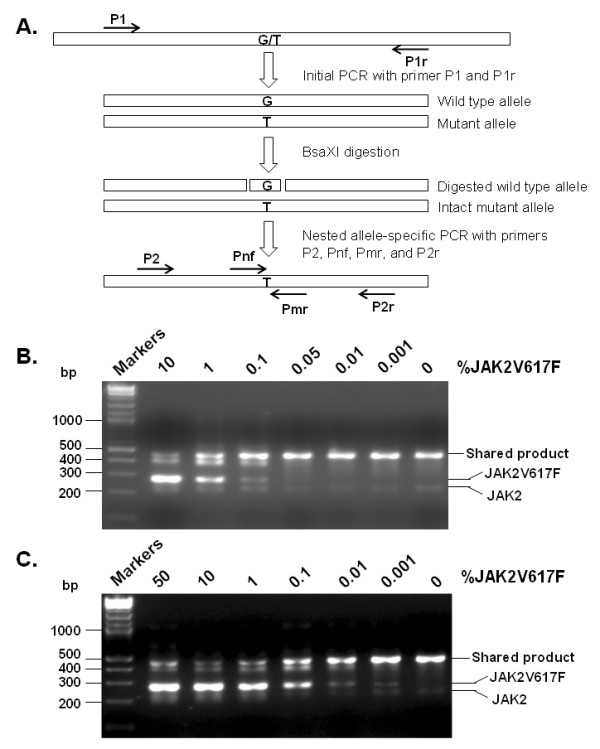
**Development of restriction fragment nested allele-specific PCR (RFN-AS-PCR), an improved method for detection of JAK2V617F**. **A**. Schematic illustration of the RFN-AS-PCR method. **B **and **C**. The sensitivities of the nested AS-PCR and RFN-AS-PCR methods were determined by using purified plasmid DNAs. Mixtures of JAK2 plasmid DNAs containing the indicated percentages of the JAK2V617F mutant were amplified with primers P1 and P1r. The PCR products were left undigested (panel B) or digested with restriction enzyme BsaX1 (panel C) and then subjected to nested AS-PCR analyses with a primer mixture containing P2, P2r, Pmr, and Pnf. The final PCR products were analyzed on 3% agarose gel, and DNA bands were visualized by staining with ethidium bromide. The positions of wild type JAK2 and mutant JAK2V617F are indicated.

AS-PCR has been widely used to detect the gene mutations [30]. This method relies on specific PCR primers to discriminate wild type and mutant alleles. It has a reported sensitivity of 0.1% to 1% mutant allele for detection of JAK2V617F [19,21,26]. Another commonly used JAK2V617F detection method is PCR-restriction fragment length polymorphism, which takes advantage of the fact that the V617F mutation disrupts a convenient BsaXI restriction enzyme digestion site. The reported sensitivity of this method is ~4% [15]. Both methods are simple and convenient since they do not require specialized equipment or expensive reagents, but each method has limitations on sensitivity and specificity. When the JAK2V617F mutation rate is low, these methods often give weak and ambiguous signals because AS-PCR is not absolutely specific, and restriction enzymes cannot digest with 100% efficiency [29]. The main problem is the overabundance of wild type allele products. We reasoned that a combination of these two methods would solve this issue and greatly enhance specificity and sensitivity. We designated the method restriction fragment nested allele-specific PCR (RFN-AS-PCR). A schematic diagram of the procedure is illustrated in Figure 1A. The method contains three steps. In the first step, a PCR fragment containing the mutation site was amplified by using a pair of outer primers. In the second step, the PCR products were treated with BsaXI enzyme to cleave the wild type allele. In this critical step, BsaXI removes from the wild type PCR product a 30 bp fragment containing the V617F mutation site, which effectively eliminates the chance that the full-length wild type product is re-generated in the subsequent PCR. In the final step, the digested PCR products were subjected to AS-PCR with nested primers.

We first employed purified plasmid JAK2 and JAK2V617F DNAs to determine the sensitivity of the method. The standards were created using a mixture of the two purified plasmid DNAs to avoid the effect of JAK2 gene copy number variation reported for the HEL cell line, which has often been used as a positive standard [18,22]. Our data demonstrated that nested AS-PCR without BsaXI digestion had a sensitivity of 0.1% (Figure 1B). With the BsaXI digestion step introduced, the RFN-AS-PCR technique increased the detection limit to 0.001% (Figure 1C), corresponding to a 100-fold enhancement.

**Figure 2:**
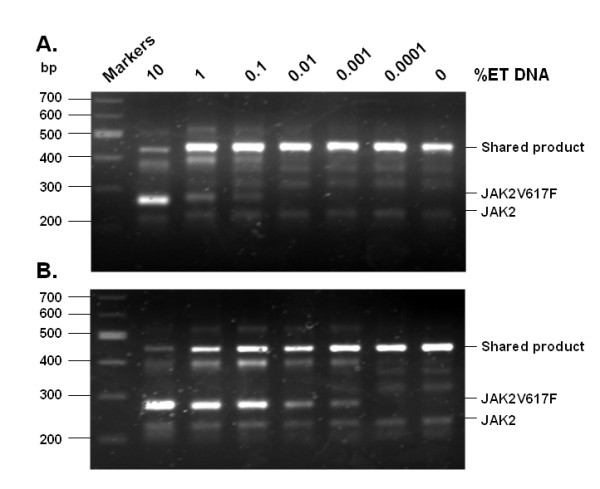
**Validation of the RFN-AS-PCR method by using mixtures of DNA samples from normal and MPN blood samples**. Blood cell DNAs from a heterozygous JAK2V617F-positive ET patient and a normal donor were mixed in the indicated proportions. Initial PCR was performed with primers P1 and P1r, and PCR products were directly subjected to nested AS-PCR (panel A) or digested with BsaXI and then subjected to nested AS-PCR (panel B). Note that the BsaXI digestion increased the detection sensitivity from 0.1% to 0.001% ET blood.

We further employed blood DNA samples to validate the sensitivity of the method. For this purpose, we mixed in various proportions a JAK2V617F-negative normal blood DNA sample and a heterozygous JAK2V617F-positive essential thrombocythemia (ET) blood DNA sample. A total of 1 μg of the DNA mixtures was used for the initial PCR, followed by nested AS-PCR with or without prior BsaXI digestion. The results are shown in Figure 2. Without BsaXI digestion, the nested AS-PCR was able to detect mixtures containing 0.1% of ET DNA. With the BsaXI digestion step, the detection sensitivity was increased to 0.001%. It should be noted that the ET patient carried a heterozygous JAK2V617F mutation and that not all white blood cells in the patient were JAK2V617F positive. In theory, the real sensitivity should be better than 0.001%.

### Identification of JAK2V617F positivity in normal blood samples

**Figure 3:**
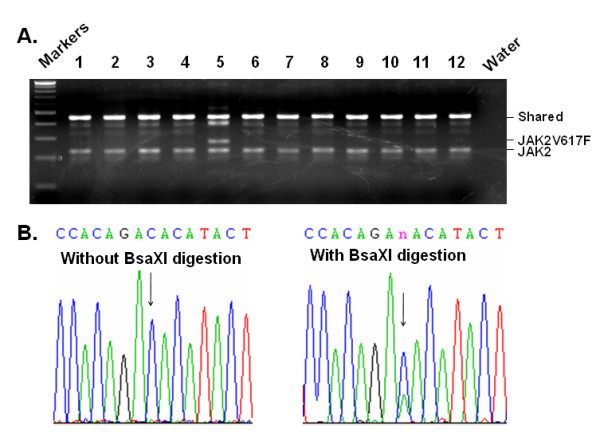
**Identification of JAK2V617F in normal blood samples**. Blood cell DNAs from normal donors were analyzed by using the RFN-AS-PCR method. **A**. A typical analysis of multiple blood samples. Sample no. 5 was identified JAK2V617F positive. **B**. Verification of the positive samples by DNA sequencing. For this purpose, the product of first round PCR with primers P1 and P1r was either left untreated or digested with BsaXI before nested PCR with primers P2 and P2r. The nested PCR products were gel-purified and subjected to DNA sequencing analysis with primer P2r. Note that without BsaXI digestion DNA sequencing failed to reveal any mutant allele, but after restriction enzyme digestion, a clear mutant allele (base A in the indicated position) was detected.

We further applied the method to detect JAK2V617F in other blood samples. In our earlier studies, by using nested AS-PCR without a BsaXI digestion, we screened over 4,000 blood samples randomly collected from a hospital population and found nearly 1% of samples to be JAK2V617F positive [19]. Although hardly any of these patients met the criteria for diagnosis of MPNs, they did have other conditions including cardiovascular diseases, which may be caused by underlying hematological disorders [19]. Intriguingly, another study using a real time PCR-based assay that combines molecular beacon probe and locked nucleic acid techniques detected JAK2V617F mutation in about 10% of healthy donors [31]. We thus decided to use our new method to analyze blood samples from healthy donors in order to evaluate the presence of the JAK2V617F mutation. We collected 105 normal blood samples from clinical laboratories. These were residual samples from routine physical exams, and the donors had an average age of 42 years (ranging from 30 to 61 years). They had normal blood cell counts and were apparently healthy. Using the traditional nested AS-PCR method without the BsaXI digestion step, not a single JAK2V617F-positive case was found in these samples (not shown). However, with our new method, we identified three positive cases. The three JAK2V617F-positive donors had ages of 45, 52, and 55, respectively. Figure 3A shows the results of a typical assay with one positive case identified. To verify the presence of the mutant allele in the samples, we performed DNA sequencing analysis. For this purpose, the initial PCR products were either treated or non-treated with BsaXI and then re-amplified with nested PCR. The nested PCR products were purified and sequenced. As shown in Figure 3B, without BsaXI digestion, no mutant allele was revealed in the sequence profile, but with BsaXI digestion, a clear signal for the mutant allele was detected. The BsaXI digestion step clearly enriches the amplicons bearing JAK2V617F. The sequencing analyses confirm that the mutation does exist and is not a PCR artifact since the C-to-A mutation which corresponds to V617F is the only mutation we detected in the 453 bp DNA fragment amplified by PCR. Although the number of JAK2V617F-positive cases is relatively small for statistical conclusion, the data suggest that JAK2V617F is present in healthy donors with normal blood cell counts. Clearly, using our method, we are able to identify JAK2V617F-positive samples that could not be detected by less sensitive methods.

### Impact of the RFN-AS-PCR method on future research and applications

By combining nested AS-PCR and specific restriction enzyme digestion, we have developed a highly sensitive method dubbed RFN-AS-PCR for detection of JAK2V617F. With a sensitivity of about 0.001% mutation rate, the highest sensitivity for JAK2V617F detection reported so far, the method is simple, quick, and inexpensive, not requiring specialized equipment and reagents. It also has all the advantages of nested AS-PCR including suitability for a very small amount of non-purified DNAs [32]. We believe that RFN-AS-PCR should serve as an important tool to screen blood samples for early diagnosis, prevention, and treatment and to study the progression of MPNs. Furthermore, the principle of this method can be applied to detection of other gene mutations.

With the RFN-AS-PCR method, we detected 3 JAK2V617F-positive cases out of 105 normal blood samples, supporting the earlier finding that a very low level of JAK2V617F is present in healthy donors [31]. The prevalence of JAK2V617F appears much higher than the incidence of MPNs, which is about 4.8 per 100,000 [33]. This does not mean that JAK2V617F is irrelevant to MPNs as one may suspect, but rather suggests that the JAK2V617F mutation is a very early molecular event. MPNs are chronic diseases mainly affecting the elderly with an average onset of 55 years [1]. In MPN patients, JAK2V617F burden, that is, the percentage of JAK2V617F in the total JAK2 DNA, varies. Some patients may have a JAK2V617F burden close to 100% while others may have a relatively low level of 5% [7]. Although there is no direct correlation between JAK2V617F burden and the elevation of blood cell counts, it is generally believed that a higher JAK2V617F burden reflects advanced status of the MPN disease. PMF is considered to be the most severe form of MPNs and thus has the highest JAK2V617F burden on average, while ET, the least severe form of MPNs, has the lowest JAK2V617F burden [34,35]. In accord with the fact that MPNs are chronic blood diseases, JAK2V617F does not cause malignant transformation like many other oncogene products but rather causes a progressive increase of blood cells. For those individuals with a very low percentage of cells with JAK2V617F, it may take many years to show a clear MPN symptom, and some may never reach the stage before they die of other diseases. It is also likely that JAK2V617F-bearing cells may stay dormant until they are triggered to proliferate by certain environmental factors.

The presence of JAK2V617F may indicate future development of other diseases. In an earlier study using a nested AS-PCR method without the BsaXI digestion step, we screened over 4,000 blood samples randomly collected from hospital patients and found nearly 1% of samples to be JAK2V617F positive. Hardly any of these patients met the criteria for diagnosis of MPNs, but many of them had other conditions including cardiovascular diseases, which may be the result of underlying hematological disorders [19]. The majority of these patients had a JAK2V617F burden of less than 5%. In theory, the presence of a small percentage of JAK2V617F-positive cells may take a long time to produce a full-scale MPN phenotype, but may be sufficient to cause vascular damage and thereby trigger heart disease. In any case, relevance of JAK2V617F positivity with cardiovascular disorders warrants further investigations.

Discovery of JAK2V617F represents a milestone in the MPN field [2-7]. Because of its pathogenicity and constitutive activation nature, JAK2V617F represents an obvious target for therapeutic drug development. Indeed, many potent JAK2 inhibitors have been identified, and some have gone through clinical trials, but most of these studies produced generally disappointing outcomes [36,37]. However, we do not believe this diminishes the pathogenic role of JAK2V617F in causing MPNs, as the unsatisfactory clinical results may be largely due to the selection of patients, who were often at a very late stage of MPN development. We believe that early treatment may be the key, as seen in the effective treatment of chronic myeloid leukemia with BCL-Abl inhibitors [38]. In this regard, our study provides a powerful tool to detect JAK2V617F positivity at a very early stage.

## Conclusions

We have developed a highly sensitive method dubbed RFN-AS-PCR for detection of JAK2V617F. This method has a sensitivity of 0.001% mutation rate, the highest reported so far. The method is simple, quick and versatile. With this method, we were able to detect the presence of a low level of JAK2V617F in a small fraction of normal blood samples. Our study provides a powerful tool to detect JAK2V617F positivity at a very early stage and should have major implications in diagnosis and prevention of MPNs and other diseases that may be affected by JAK2V617F.

## Methods

### Sample collection and DNA extraction

De-identified normal and MPN blood samples were collected from local clinical laboratories. The samples were all residual blood from complete blood cell count tests. The normal blood samples were obtained from healthy donors subjected to routine physical exams. Institutional review board approval was obtained before these samples were collected and analyzed. Genomic DNAs were purified by using the phenol/chloroform method following proteinase K digestion of total white blood cells. For each PCR reaction described later, up to 1 μg total DNA was used.

### Plasmid DNA standards derived from wild type JAK2 and JAK2V617F

As described previously [19], 521-bp DNA fragments from genomic DNAs containing wild type and V617F mutation JAK2 were amplified with primers P1 (5'- GATCTCCATATTCCAGGCTTACACA) and P1r (5'- TATTGTTTGGGCATTGTAACCTTCT) and then cloned into the pBluescript KS vector (Stratagene). Plasmid DNAs were purified from *E. coli *cells by using the PureLink™ HiPure Plasmid DNA Purification Midiprep kit from Invitrogen. DNA concentrations were determined by measuring absorbance at 260 nm. Purified JAK2 and JAK2V617F plasmid DNAs were mixed at different proportions and diluted to 20 μg/ml with 0.2 mg/ml salmon sperm DNA as a carrier, and 1 μl of the DNA sample mixtures were used for PCR analysis described below.

### Initial PCR, BsaX1 restriction enzyme digestion, and nested AS-PCR

A schematic illustration of the RFN-AS-PCR method is provided in Figure 1A. In brief, an isolated plasmid DNA or blood cell DNA was used as a template for initial PCR with primers P1 and P1r. The PCR was run with Taq DNA polymerase for 35 cycles with each cycle consisting of 94°C for 20 sec, 60°C for 20 sec, and 72°C for 30 sec. The PCR products were then digested in a 10 μl reaction mixture containing 1 μl PCR products and 0.4 unit of BsaX1 (New England BioLab) at 37°C for 2 hr. The digested PCR products were further subjected to AS-PCR with nested primers P2 (CCTCAGAACGTTGATGGCA) and P2r (ATTGCTTTCCTTTTTCACAAGA) and allele-specific primers Pnf (AGCATTTGGTTTTAAATTATGGAGTATATG) and Pmr (GTTTTACTTACTCTCGTCTCCACAAAA). The PCR was run for 35 cycles with each cycle consisting of 94°C for 20 sec, 60°C for 20 sec, and 72°C for 20 sec. The final PCR products were resolved on 3% agarose and visualized with ethidium bromide staining. To ensure no cross-contamination occurred, control experiments with water replacing DNA samples were routinely performed and filter tips were used throughout. For sequencing verification, PCR products were gel-purified and then analyzed by using an ABI3730 capillary sequencer. Each of the above experiments was repeated at least three times with consistent results.

## List of abbreviations

AS-PCR: allele-specific polymerase chain reaction; ET: essential thrombocythemia; MPN: myeloproliferative neoplasm; PMF: primary myelofibrosis; PV: polycythemia vera.

## Competing interests

The authors declare that they have no competing interests.

## Authors' contributions

AHZ designed and performed the research experiments; RG designed and supervised the research; ZJZ designed the experiments. All authors wrote, read, and approved the manuscript.
